# Identification of wheelchair seating criteria in adults with neuromuscular diseases: A Delphi study

**DOI:** 10.1371/journal.pone.0290627

**Published:** 2023-09-08

**Authors:** Elise Dupitier, Manon Voisin, Caroline Stalens, Pascal Laforêt, Samuel Pouplin

**Affiliations:** 1 UR2020 ERPHAN, Paramedical Research Team in Neuromuscular Disability, Paris—Saclay University, University Hospital Site of Raymond Poincaré, Garches, France; 2 UFR Simone Veil-Santé, UMR 1179 End-iCap, Paris—Saclay University, University Hospital Site of Raymond Poincaré, Garches, France; 3 Medical Department, AFM-Téléthon, Evry, France; 4 National Invalid Institution, Paris, France; Fondazione Policlinico Universitario Gemelli IRCCS, ITALY

## Abstract

**Background:**

Adults with neuromuscular diseases like spinal muscular atrophy or Duchenne muscular dystrophy require full-time use of a wheelchair (WC) and perform all activities of daily living in a sitting position. Optimal configuration of the WC and seating system is essential to maintain the health and quality of life of users. However, few recommendations for configuration exist. The aim of this study was to identify and select 10 WC seating criteria that ensure an optimal sitting posture for health and quality of life.

**Methods:**

A four round Delphi method was used to collect the opinions of WC users and health professionals (HP), separately. First, the HP were asked if they believed that different criteria would apply to each disease. Then the HP and SMA II and DMD WC user experts responded to electronic surveys in 4 rounds.

**Results:**

Overall, 74 experts took part: 31 HP, 21 WC users with SMA II and 22 WC users with DMD. In total, 52% of HP believed that different criteria would apply to each disease. Ten criteria were identified by the HP for SMA II and 10 for DMD. Of the 40 criteria selected, 30 (75%) were common to each panel. Six topics were similar across panels: comfort, access to the joystick, prevention of pain, stability, pressure management and power seat functions. However, power seat functions did not reach consensus between HP and WC users (30–33% of agreement for HP and 93–100% for the WC user panels, p < 0.001).

**Conclusion:**

Adults with SMA II and DMD had similar WC seating needs. Therefore, the same recommendations can be applied to these groups. Further research is necessary to understand the impact of cost on the prescription of power seat functions by health professionals.

## Introduction

Neuromuscular diseases (NMD), such as spinal muscular atrophy type II (SMA II) and Duchenne muscular dystrophy (DMD), are rare genetic diseases. SMA is an autosomal recessive neurodegenerative disorder caused by a defect in the survival motor neuron (*SMN1*). Type II is an intermediate form, with a prevalence of 2 per 100 000 [1]. DMD is an X-linked disorder that results from a mutation in the dystrophin gene, with a prevalence of 9.9 per 100 000 [1]. The fulltime use of a powered wheelchair (WC) is necessary for people with SMA II from the normal age of walking achievement [2] and for people with DMD from the age of 10 to 13 years [3]. Before the advent of new therapies for SMA, i.e., in 2017 in France, children with SMA II who could sit independently lost this ability, along with head control, in adulthood. Similarly, adults with DMD lose independent sitting and head control over time. In addition to loss of ambulation, these diseases cause scoliosis and muscle contractures [2,4], which impact on sitting posture, comfort in the WC [5] and performance of activities of daily living. Scoliosis is a common consequence of other neuromuscular disorders such as spinal cord injury, cerebral palsy and spina bifida [6]. However, the prevalence of scoliosis is particularly high in DMD and SMA: 60–90% in SMA and 90% in DMD, without glucocorticoid therapy [2,7]. Most affected individuals undergo instrumented spinal surgery to correct or prevent worsening of scoliosis. Pelvic alignment disorders, and trunk and head collapse are also frequent. Intrinsic myotendinous structural changes and extrinsic factors often cause joint contractures around the hips, knees, elbows and wrists in both SMA and DMD [8]. Furthermore, prolonged WC use causes other comorbidities such as chronic pain and pressure injuries [9–13]. Pain in DMD is mainly ischial or around spinal deformity sites [9,11]. In SMA II, pain is mainly in the neck, back and legs. Poor sitting posture is considered as the largest pain exacerbating factor and changing position as the best pain relieving strategy [14,15]. The benefits of WC use on mobility are undeniable; however, the large number of hours spent in a sitting position each day has adverse effects on health and quality of life [16,17]. For example, in DMD, a high risk of pressure injuries, which increases with age, has been reported [9,11]. The type of cushion, backrest, seating components such as posterior or lateral supports or belts, as well as the WC size and parameters can either improve sitting tolerance and social participation [18,19] or can actually have a negative impact on the person’s life [20,21]. Three interventional studies have been published on the effects of wheelchair seating, 2 in DMD [22,23] and 1 in SMA II [24]. They report contradictory and sometimes controversial results for the influence of postural devices on pulmonary function in DMD, whereas a positive effect was found for SMA II. They also showed that postural support devices, like an individually selected cushion, backrest, upper limb and trunk support and headrest, improve upper limb function in both diseases and improve posture in DMD.

International recommendations for SMA [2] and DMD [3] provide general guidance about wheelchair seating with references to custom seating systems, power seat functions and WC standing devices. However, more clinically-orientated guidelines are needed for rehabilitation teams to supplement guidelines that have been published in the grey literature for rare, genetic NMDs [25,26].

Our long-term aim is to develop a prognostic score that identifies the need to change the wheelchair seating system for a given individual; however, a clear identification of the main criteria that ensure the health and quality of life of WC users with SMA II and DMD is first required. Furthermore, to our knowledge, the needs and expectations of adults regarding WC seating have never been expressed through a consensus method in which the participants are exclusively users, with no influence from HP. Furthermore, user opinions have never been compared with the opinions of health professionals (HP) who may be influenced by the French social and economic system. The aim of this study was to identify and select 10 WC seating criteria that maintain health and quality of life according to the opinions of WC users (SMA II and DMD) and HP. We also wished to determine if the criteria identified were similar between WC users and HP; we hypothesized that they would be similar. Finally, we wanted to describe the profiles of the HP experts and the WC users and to determine if they shared the same characteristics.

## Methods

### Study design

Before beginning the study, we sent a preliminary questionnaire to the HP who were included, asking if they thought that the same seating criteria could be applied to people with DMD and SMA II. A slight majority, 52%, responded that different criteria would apply; therefore, we constituted two different panels of wheelchairs users.

We used the Delphi method and included 3 panels of experts: HP, adult WC users with SMA II and adult WC users with DMD. We conducted the study according to the following recommendations: anonymity of experts between each other, iteration of questionnaires and controlled feedback [27,28]. A classic Delphi technique with 4 successive rounds was used.

The study was conducted in two phases: Phase 1 (March 2020 to June 2021) involved the HP panel and Phase 2 (July 2021 to February 2022) involved both panels of WC users.

Data were collected from the HP using electronic questionnaires created with “Drag’n Survey”, an online survey creation and management software. This part of the study was approved by the ethics committee of the Versailles Saint Quentin University (CER-Paris-Saclay-2020- 054).

The information technologists of the health data host “multi-health” designed a data collection platform with interactive forms that was sent as a link to the two panels of wheelchair user experts. This part of the study was approved by the ethics committee “Sud-Est VI” of the university hospital center of Clermont Ferrand N°2020-A02854-35.

### Recruitment of experts

The panels were composed of people with experience in the field studied and was not intended to be representative. However, special attention was paid to ensuring the geographical homogeneity of experts to avoid an over representation of possible regional practices. We aimed to recruit a minimum of 30 experts according to recommendations [28].

#### HP experts

HP experts were selected within the French neuromuscular network and French seating and mobility centers network. The inclusion criteria were as follows: physical and rehabilitation medicine physician, occupational therapist, or orthopedic surgeon, at least 5 years of experience in NMD and WC seating, and ideally having published or communicated in a conference on these topics. These 3 categories of professionals were identified because of their direct implication in the sitting posture management of adult WC users and in the selection and prescription process of specialized seating and WC in France. AFM-Téléthon, a national association for neuromuscular diseases and Positi’F, a French association recognized in France to assemble health professionals in seating clinics, helped us to identify potential experts. An email was sent to potentially eligible experts and the inclusion criteria of those who responded positively were verified by telephone.

#### Wheelchair user experts (DMD and SMA II)

Two panels were constituted: SMA II adult WC users and DMD adult WC users.

Because most of these users live at home, we recruited through AFM-Téléthon, which has support networks for people with a disability living at home. We sent emails to the 16 regional support teams. In parallel, we request 30 rehabilitation teams specialized in the follow up of adults with NMD. An announcement was also published on the AFM-Téléthon website. All these networks covered the whole country. Inclusion criteria were as follows: having used a power wheelchair for at least 5 years, being a full-time user (more than 4 hours a day) and previously or currently followed by a rehabilitation physician and an occupational therapist for wheelchair seating. After agreeing to participate or showing interest to the physician, occupational therapist or professional advisor at the AFM-Téléthon, individuals were invited to send an email to the investigators. The inclusion criteria were then verified by email exchange.

### Data collection

**Fig 1:**
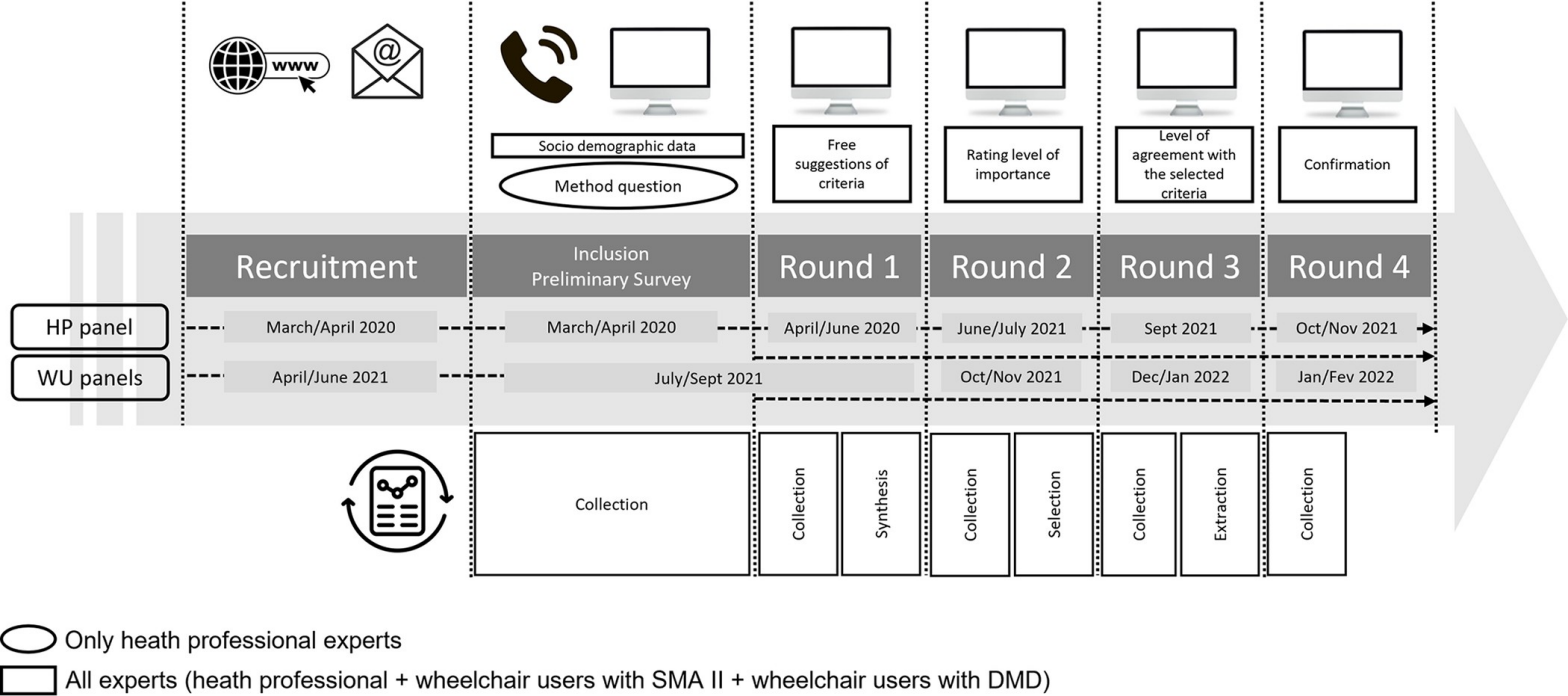
Survey timeline.

The survey was conducted in four rounds (Fig 1). Data from each round were analyzed by two occupational therapists specialised in wheelchair seating for people with NMD. One had 16 years of experience and the other 1 year. Both were familiar with the vocabulary and technical terms relating to NMD and seating. Throughout the study, the investigators had access to information that could identify individual participants to ensure the timeliness of the responses.

#### Round one

Questionnaire n°1 consisted of 1 open-ended question: “Please suggest between 6 and 10 criteria required for good positioning in the wheelchair (ie, to maintain the health and quality of life of users)”. HP were asked to provide separate criteria for people with SMA II and DMD and to use a verb for each criterion suggested. The WC user panels were asked the same question but only for their own disease.

#### Round two

Questionnaire n°2 presented a synthesis of all the criteria suggested in round one, after removal of duplicates. The HP panel were asked to separately analyze the criteria for people with SMA II and people with DMD and the WC user panels were asked to analyze the criteria relating to their own disease. The aim of this round was to select the ten most important criteria by ranking all the criteria according to their importance on a Likert scale of 1 to 9 points (1 = “not at all important” and 9 = “extremely important”). The ratings from all participants were then averaged and the 10 best were identified. In the event of a tie, the standard deviation was considered.

#### Round three

Questionnaire n°3 presented the 10 criteria obtained from round two. The experts were asked rate their level of agreement with the selected criteria using a Likert scale of 1 to 4 points (1 = “strongly disagree” and 4 = “strongly agree”).

#### Round four

Questionnaire n°4 presented the distribution of responses from the 3 panels along with the expert’s own responses. The experts were asked to confirm or modify their choices using the same 4-point Likert scale as in round three.

We only selected criteria that obtained at least 70% consensus in round 4. The percentage consensus was calculated by taking account the number of experts in each panel who agreed or strongly agreed with the criteria selected.

### Statistical analysis

To characterize the 3 expert panels, continuous outcomes were reported as mean (SD) and categorical variables were reported as number (percentage). Categorical variables were compared using a Chi square test or Fisher’s exact test. To compare the means of continuous variables between the 3 panels, a one-way Anova was performed. To compare the means of outcomes between two panels, a Student t test was used. There was no management of missing data.

All statistical tests were two-sided, with p-values < 0.05 denoting statistical significance. All analyses were performed with JASP 0.14.1.0.

## Results

### Participants

**Fig 2:**
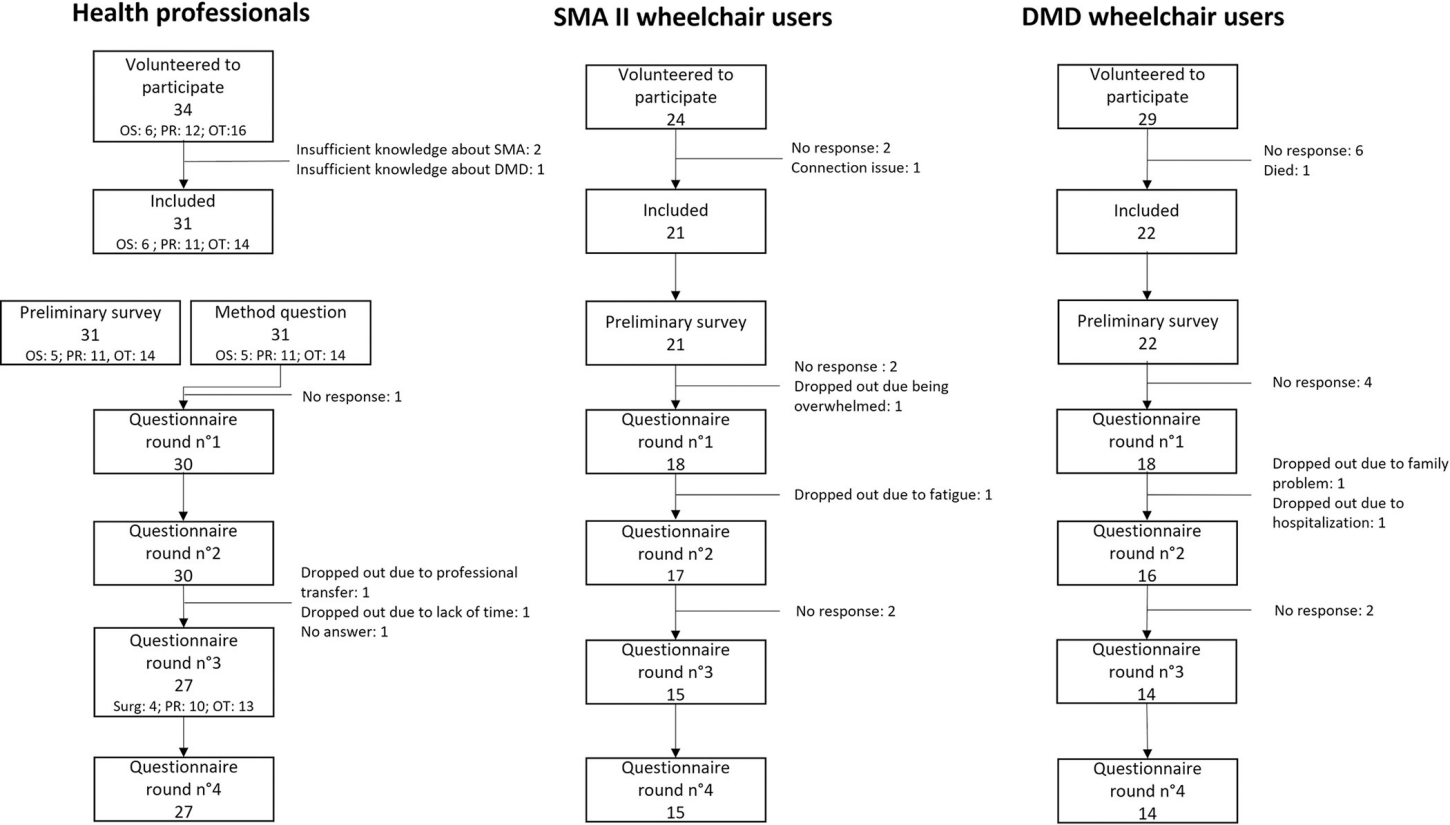
Number of participants in each survey round.

Of the 87 experts who initially agreed to participate, 74 actually participated (see flow chart in Fig 2).

The panel of HP experts was composed of 31 participants: 14 occupational therapists (OT), 11 rehabilitation physicians (RP) and 6 orthopedic surgeons (OS). The SMA II WC user panel was composed of 21 participants and the DMD panel of 22 participants.

The 87 experts were from the 13 main regions of France; however, the island of Corsica and overseas territories were not represented. Considering each panel individually, between 2 and 4 main regions were for each (2 for SMA II WC users, 4 for DMD WC users and 3 for HP panel).

Fig 2 presents the flow chart of participants in each round.

A total of 56 experts participated in round 4, 27 HP (13 OT, 10 RP, 4 OS), 15 experts with SMA II and 14 with DMD. Therefore, the attrition rate was 13% for the HP, 28.5% in SMA II WC panel and 36.5% for the DMD WC user panel. There was no loss of homogeneity within each panel.

#### Health professional experts

The mean (SD) age of the HP panel was 48.4 (10.3) years. The experts had a mean 16.7 (9.7) years of experience in NMD and 17.1 (10.3) years in wheelchair seating. Mean self-assessed rating of their level of expertise in wheelchair seating in NMD was 4.6 (1.1) on a 7-point scale (1 to 7). The majority (90%) worked in a university hospital within a specialized NMD team (80%). More than half (71% of OT and 54.5% of RP) also worked within a wheelchair specialist team.

More details are provided in Table 1.

**Table 1:** Characteristics of the health professional panel.

	Occupational therapists n = 14	Rehabilitation Physicians n = 11	Orthopaedic surgeons n = 6	Total N = 31	p-value
**Sex**, n (%) *Male* *Female*	3 (21%) 11 (79%)	4 (36%) 7 (65%)	6 (100%) 0 (0%)	13 (42%) 18 (58%)	0.004*
**Age, mean (SD)**	42.8 (5)	50 (9.1)	58.5 (13.9)	48.4 (10,3)	0.003*
**No. of years of experience in NMD mean (SD)**	13.4 (4.2)	15.5 (7.7)	26.8 (15.6)	16.7 (9.7)	0.010*
**No. of years of experience in WC seating mean (sd)**	14.1 (7.6)	16.4 (8.2)	25.2 (15.7)	17.1 (10.3)	0.081
**Place of practice,** n(%) *University hospital* *NMD Team* *WC seating team* *PI team* *Pain team*	11 (79%) 10 (71%) 10 (71%) 5 (36%) 1 (7%)	11 (100%) 9 (89%) 6 (54%) 2 (18%) 3 (27%)	6 (100%) 6 (100%) 0 (0.0%) 0 (0.0%) 0 (0.0%)	28 (90%) 25 (81%) 16 (52%) 7 (23%) 4 (13%)	0.402
**Additional qualifications** (highest level), n (%) *UD* *PhD* *MSc*	4 (29%) 1 (7%) 0 (0%)	5 (45%) 3 (27%) 1 (9%)	1 (17%) 4 (68%) 0 (0.0%)	10 (32%) 8 (26%) 1 (3%)	0.123
**Knowledge pathways about NMD,** n(%) *A lot from experience* *A lot from scientific littérature* *A lot from continuing education* *A lot from professional training*	13 (93%) 3 (21%) 3 (21%) 2 (14%)	8 (73%) 5 (45%) 4 (36%) 2 (18%)	6 (100%) 5 (83%) 1 (17%) 1 (17%)	27 (87%) 13 (42%) 8 (26%) 5 (16%)	0.190 0.090 0.519 0.994
**Knowledge pathways about seating,** n (%) *A lot from experience* *A lot from scientific littérature* *A lot from continuing education* *A lot from professional training*	14 (100%) 3 (21%) 5 (36%) 1 (7%)	9 (82%) 3 (27%) 2 (18%) 1 (9%)	6 (100%) 1 (17%) 1 (17%) 1 (17%)	29 (93%) 8 (26%) 8 (26%) 3 (10%)	0.143 0.879 0.465 0.221
**Involvement in publications,** n (%)	6 (42%)	4 (37%)	2 (33%)	12 (39%)	0.281
**Presenting at conferences,** n (%)	10 (71%)	8 (73%)	4 (67%)	22 (71%)	0.934
**Level of expertise** (self-assessment) §	4.7 (1.1)	4.3 (1.0)	4.8 (1.3)	4.6 (1.1)	0.076

No. = Number; NMD = NeuroMuscular Diseases, WC = Wheelchair, PI = Pressure Injury, UD University Diploma.

* significant result.

§ Scale from 1 to 7, 1 = very low, 7 = very high.

There were no significant differences between the 3 groups of HPs except that OS (83%) read more NMD literature than the others (21% for OT and 45% for PR), p = 0.035. They practiced in the same types of centers, except that the surgeons were not integrated within seating clinics.

#### Wheelchair user experts (Table 2)

The mean age of the SMA II panel was 37.3 (11.6) years and they had used a wheelchair for a mean 29.0 (8.0) years. The mean age of the DMD panel was 32.1 (8.4) years and they had used a wheelchair for 22.0 (8.0) years. Mean self-assessment rating of the level of expertise was 4.8 (1.1) (/ 7 points) for the SMA II panel and 4.9 (1.2) for the DMD panel.

**Table 2:** Characteristics of the wheelchair userpanel.

	SMA II users n = 21	DMD users n = 22	p-value
**Sex**, n (%) *Male* *Female*	5 (24%) 16 (76%)	22 (100%) 0 (0%)	<0.001*
**Age**, Mean (SD)	35.9 (11.5)	30.9 (8.4)	0.111
**Level of education** *Primary school* *End of middle school* *End of high school* *Two years after high school* *Bachelor’s degree* *Master’s degree* *PhD*	1 (5%) 5 (24%) 5 (24%) 3 (14%) 3 (14%) 3 (14%) 1 (5%)	3 (14%) 5 (23%) 5 (23%) 1 (4%) 6 (27%) 2 (9%) 0 (0%)	0.315
**Employment**, n (%) *Full time active* *Part time active* *None* *Student*	4 (19%) 2 (9%) 13 (62%) 2 (9%)	1 (4%) 2 (9%) 16 (73%) 3 (14%)	0.051
**No. of years of WC use**, n (%)	29.0 (8.0)	22.0 (8.0)	0.010*
**Age at first WC, Mean (SD)**	7.4 (5.3)	10.1 (1.7)	0.034*
**Hours in wheelchair per day**, Mean (SD)	12.5 h (3)	11.1 h (2.4)	0.135
**Time since last seating consultation** (months), Mean (SD)	44.1 (67.2)	33.9 (34.3)	0.530
**Time since last modification to WC** (months), Mean (SD)	40.6 (41.3)	28.7 (23.9)	0.260
**Type of WC currently used**, n (%) *Power WC* *With reclining backrest* *With power tilt in space* *With power elevated legrest* *With power tilt forward* *With power standing*	21 (100%) 20 (95%) 20 (95%) 15 (71%) 11 (52%) 6 (33%)	22 (100%) 20 (91%) 20 (91%) 19 (86%) 14 (64%) 7 (32%)	0.578 0.578 0.116 0.327 0.817
**Power seat functions often/ very often used,** n (% of users with power seat function) *Recline* *Tilt in space* *Elevated legrest* *Tilt forward* *Standing in wheelchair*	18 (90%) 14 (70%) 9 (60%) 4 (36%) 2 (40%)	16 (80%) 10 (50%) 10 (53%) 4 (29%) 1 (14%)	0.405 0.179 0.148 0.230 0.634
**Type of cushion currently used**, n (%) *Foam* *Air* *Gel (with part of foam)* *Custom seating*	11 (52%) 5 (24%) 3 (14%) 2 (9%)	8 (36%) 12 (54%) 2 (9%) 0 (0%)	0.099
**Level of expertise** *(self-assessment)§*, Mean (SD)	4.8 (1.1)	4.9 (1.2)	0.997

No. = Number, WC = Wheelchair.

* significant result.

§ Scale from 1 to 7, 1 = very low, 7 = very high.

### Consensus

#### Round one

For SMA II, 151 different criteria were suggested by the HPs and 86 by the SMA II WC users.

For DMD 158 criteria were suggested by the HPs and 82 by the DMD WC users.

A list of the criteria is presented in Appendix 1 in S1 File.

#### Round two

The 10 criteria with the highest mean importance ratings (7.6 to 9.0/9.0 points) for each expert panel and the two diseases are presented in Table 3. To facilitate the presentation of the results in this article, the experts’ sentences have been summarized using keywords (KW). The keywords were not used during the different rounds. The keywords are words cited by the experts themselves. They reflect criteria that are directly related to wheelchair seating and sitting posture and are specific and measurable.

**Table 3:** The 10 criteria suggested by each panel (HP panel for SMA II WC users, SMA II WC users, HP panel for DMD WC users and DMD WC users). The table presents the highest importance ratings in round two and the level of consensus in rounds three and four. Criteria were classified according to their similarity across panels.

Criteria		Importancerating Round2 Mean (SD)	% Agreement Round3	% Agreement Round4
**Suggestions for SMA II by the health professional panel**				
To choose a comfortable seat	Comfort	7.9 (1.1)	89	96
To ensure mobility during activities of daily life	Mobility	7.8 (1.1)	89	96
To allow optimal access to joystick and controls whatever the position	Joystick	8.1 (1.2)	85	93
To respect the user’s anthropometric measures when choosing a WC	Seat size	8.1 (0.7)	78	89
To regularly review and question pain in the sitting position	Pain	7.9 (0.8)	78	89
To assess satisfaction, adaptation, and quality of life with the WC	QoL	7.7 (1.4)	70	85
To provide overall stability of the body	Stability	7.8 (1.2)	67	81
To suggest an upgradeable device	Upgradability	7.7 (1.2)	60	74
To relieve pressure on protruding parts of the body	Pressure	7.7 (1.3)	67	74
To suggest a reclining backrest	Power seat	7.7 (1.4)	33	30
				
**Suggestions for SMA II by the SMA II WC user panel**				
To have a suitable and comfortable cushion to be in the WC all day without buttock pain	Comfort, Pain	8.3 (1.6)	100	100
To have a cushion under the buttocks which avoid suffering, even during a long time and which provide stability	Pain, stability	8.4 (0.9)	100	100
To have a WC of the right size for your body measures	Seat size	8.3 (1.0)	100	100
Adjust the armrests & joystick to the correct height/depth to facilitate comfort and trunk stability, avoid back strain & to be able to drive the WC	Comfort, Stability, Joystick	7.7 (1.6)	100	100
To be able to easily change position without help, with joystick	Joystick	8.6 (0.7)	93	93
To use a multi-position WC (recline, elevating seat, tilt, power legrests)	Power seat	8.3 (1.4)	93	93
Do not feel the jolts too much thanks to good shock absorbers, air wheels	Comfort	7.9 (1.7)	80	80
To have a good headrest	Headrest	7.7 (1.4)	93	93
To reduce pressure under buttocks, thighs, neck and lumbar areas	Pressure	7.6 (1.3)	93	93
To be stable so that you can drive without fear of holes in the pavement, stones, grass	Stability	7.6 (1.7)	93	93
				
**Suggestions for DMD by the health professional panel**				
To prevent pain	Pain	8.0 (0.9)	89	100
To find a compromise between comfort and mobility	Comfort	8.0 (1.0)	89	100
To provide good comfort to promote long sitting durations	Comfort	8.0 (0.9)	85	96
To allow optimal access to the joystick & controls whatever the position of WC	Joystick	8.2 (0.8)	85	96
To stabilize the pelvis	Stability	8.0 (1.1)	74	93
To provide overall body stability	Stability	7.9 (1.1)	67	93
To be aware of upper limb for a maximum driving efficiency	Joystick	7.8 (1.5)	85	93
To allow visual exploration	Visual	7.9 (0.9)	67	89
To distribute the supports as the level of the pelvis	Pressure	7.8 (1.5)	63	89
To suggest a WC with power seat functions	Power seat	8.0 (0.8)	33	33
				
**Suggestions in DMD by users’ experts panel**				
To have a reclining backrest	Power seat	9.0 (0.0)	100	100
To have tilt in space and reclining backrest functions	Power seat	8.8 (0.5)	100	100
To have a suitable backrest with a good width and height for mobility and support	Stability & mobility	8.8 (0.5)	100	100
To have a suitable cushion to avoid buttock pain	Pain	8.8 (0.5)	100	100
To easily place the hand on the joystick	Joystick	8.8 (0.6)	100	100
To have a cushion to avoid pressure injury	Pressure	8.7 (0.7)	100	100
To be surrounding by qualified health team in order to get the best advice and improve the WC set-up	Team	8.6 (0.8)	100	100
To choose the right cushion for good seating comfort	Comfort	8.5 (0.8)	100	100
To adjust the armrests so that the shoulders are relaxed and the muscles are not overstretched	Muscle prevention	8.5 (0.8)	100	100
To have a well-adjusted headrest to be adequately supported	Headrest	8.4 (0.9)	100	100

Out of 40 criteria (10 per group), 30 (75%) were similar for DMD and SMA II.

Six keyword topics were common across the 4 criteria groups (i.e. those generated by the HP for SMA II, HP for DMD, SMA II WC users and DMD WC users, Table 3) and used several times by almost all panels: comfort (cited 7 times out of 40, rated between 7.7 and 8.5), stability (cited 7 times, rated between 7.6 and 8.8), joystick (cited 6 times, rated between 8.1 and 8.8), pain (cited 5 times, rated between 7.9 and 8.8), power seat functions (cited 5 times, rated between 7.7 and 9.0) and pressure (cited 4 times, rated between 7.6 and 8.7).

Seat size was commonly cited by the HP panel for SMA II and by the user experts with SMA II (rated between 8.1 and 8.3).

#### Round three

In round three, the consensus for the 10 criteria varied from 67% to 100%, except in the HP panel for two criteria: “to suggest a reclining backrest” for SMA II (KW: power seat) (33% consensus) and “to suggest a WC with power seat functions” for DMD (KW: power seat) (33%) (Table 3) despite respective importance ratings of 7.7 (1.4) and 8.0 (0.8)/ 9 points during round two.

Four of the SMA II criteria reached 100% consensus with the SMA II WC user panel and all of the DMD criteria reached 100% consensus in the DMD WC user panel.

#### Round four and selected criteria

Consensus increased slightly from round three to four, to between 74% and 100%. This was with the exception of the same two criteria in the HP panel as round three: “to suggest a reclining backrest” (KW: power seat) for SMA II, which decreased to 30% and “to suggest a wheelchair with power seat functions” (KW: power seat) for DMD, which remained the same as in round 3: 33%.

Therefore, 10 criteria were considered to have achieved consensus in both the WC user panels and 9 in the HP expert panel for SMA II and for DMD. The statistical comparisons all showed p>0.1.

Among the 6 criteria common to the 4 criteria groups, comfort had the strongest consensus with 96 to 100%, according to the different panels, followed by access to the joystick, between 93% and 100% then pain with 89% to 100%, stability with 81% and 100%, pressure between 74% and 100% and power seat functions with 30% and 100% (Table 3).

The difference in the level of agreement between the HP panel (30–33% of agreement) and the WC user panels (93–100% of agreement) about seat power functions in SMA II and in DMD was significant, p < 0.001. The disagreement within the HP panel may have been driven by the occupational therapists since 50 and 75% (for SMA and DMD, respectively) of orthopedic surgeons and 40 and 50% of rehabilitation physicians agreed regarding the prescription of these functions. In contrast, only 8 and 15% of occupational therapists (p = 0.055 for SMA II; p = 0.074 for DMD) agreed on this point.

## Discussion

The aim of this study was to identify and select 10 WC seating criteria that preserve health and quality of life according to the opinions of WC users (SMA II and DMD) and HP. We also wished to determine if the criteria identified were similar between WC users and HP and to describe the profiles of the experts. Six criteria were similar for both diseases and were selected by all the panels: comfort, pain prevention, access to the joystick, stability, pressure distribution and power seat functions. Other criteria were selected by only 1 or 2 panels. Self-assessment of panel expertise was high and the sociodemographic characteristics of the panels were homogeneous.

### Criteria selected and similarities across panels

From round two, 6 criteria were similar across all the panels and several were cited more than once by each panel. A total of 30 of the 40 keywords (75%) were common to the two diseases. The results of this survey of both WC users and health professionals are important because they show that the same WC seating recommendations can be made for people with different types of NMD. Even if this consensus was made for adult users, the same main general criteria can be found in parts of practical guidelines about children and adolescents [25,26]. Moreover, these issues can apply to many different WC users, for example, those with SCI. Seating practice guidelines for individuals with different pathologies suggest the aims are the same across pathologies [29]. Studies have shown that comfort in the WC [30–34], pain [31,35–40], stability [30,35,41,42], pressure [31,43–45], access to the joystick whatever the sitting position [46,47], and the use of power seat functions [17,32] are issues for people with chronic stroke, amyotrophic lateral sclerosis, traumatic brain injury, spinal cord injury, cerebral palsy, multiple sclerosis, and elderly people with disability. This contrasts with the fact that before beginning the survey 52% of the HP panel believed that different criteria would apply to each disease. The slight majority highlighted the disagreement between HP regarding this question. The use of the Delphi method, which avoids influence between experts, allowed a clearer answer to emerge. Most of the OS (83%) believed that the criteria would differ between the two pathologies, whereas the OT and RP were more divided (43% and 45% respectively). The reason for these differences is uncertain. Medical training often involves reasoning by pathology. However, both rehabilitation physicians and occupational therapists base their practice on the international classification of functioning, disability, and health model. Moreover, although surgeons are prescribers of seating systems in France, they are not usually involved in seating assessments. None of the surgeons who participated were integrated in seating clinics. This survey is a first step to confront current opinions in France with other European or even international practices.

#### Comfort and pain

These 2 criteria are discussed together because they are closely related concepts. Discomfort is an “unpleasant body feeling or sensation that can be divided into pain or other unpleasant physical feelings or sensations such as fatigue, sleeplessness, shortness of breath, or thirst” [48]. The International Association for the Study of Pain describes pain as “an unpleasant sensory and emotional experience associated with actual or potential tissue damage” [49].

Studies have reported a prevalence of WC related pain or discomfort between 31% and 41% in WC users with DMD and 86% in WC users with NMD and muscular diseases [9,11,13,38]. The topography of the pain is mainly the posterior thigh, the ischial areas and lateral thoracic areas [9,11], with a higher prevalence for worse disability stages. The WC users with SMA II and the HP for DMD used these keywords in relation to the long daily duration of sitting. Indeed, the WC user panel spent an average of 11.1 to 12.5 hours sitting per day. The main outcome measures for pain and discomfort are visual analog and numeric scales; specific tools have also been developed, like the tool assessment for wheelchair comfort (TAWC) [30]. However, we found no studies that identified a cut off score above which the user or HP should react to improve the situation. In practice, clinicians agree that no discomfort or pain should be induced by WC use.

These findings demonstrate the importance of considering comfort and pain in decisions regarding wheelchair seating. These issues should be systematically addressed in consultations by thoroughly questioning the user. This is particularly relevant since pain is frequently underestimated by professionals and under-expressed by users despite being a major issue for WC users [38,50].

#### Access to the joystick in all sitting positions

This criterion was in second position in terms of the number of citations and consensus rating. Permanent access to the joystick is crucial because the joystick allows freedom of movement and the independence of the WC user. The joystick is used to drive the WC, to operate the power seat functions and to control the environment [51]. In short, access to the joystick is essential for the autonomy of power WC users in daily life. The position of the joystick and the electronic functions are extremely important [52]. However, access to the joystick is also guaranteed by the stability of the whole body in the chair, as well as the upper limb, in the case of proximal impairment like in SMA II and DMD. Movements of the armrest must follow those of the backrest to avoid losing the joystick. The HP suggested adding stops at the elbow and/or forearm to secure the position of the hand on the joystick, whatever the WC position, for people with SMA II (criterion 34 in round one) and for DMD (criterion 33, Appendix 1 in S1 File). The seating assessment includes the choice of the most suitable joystick. Progression of the disease and condition requires regular review of the components of access to the joystick. A study in people with amyotrophic lateral sclerosis (ALS) showed that 85% of respondents to a survey were satisfied with their armrest at 1 month, whereas at 6 months, this proportion had reduced to 70% [53]. Disease progression must be considered because an unsuitable joystick or unsuitable joystick position can lead to adverse postural behaviours, something which we regularly observe in the seating clinic. It is essential to ensure that joystick access is enabled by the seating systems and is maintained during the use of powered seat functions.

#### Stability

Stability was also frequently cited, with a high level of consensus.

Stability is an important component of daily WC use because it determines access to the joystick and to the environment to optimize performance during activities [54]. Stability also contributes to confidence in the use of the WC. Stability depends on the individual’s postural control and the support provided by the seating system. In SMA II and DMD, postural control in sitting is poor because of the generalized muscle weakness [55], and stability is essentially provided by the seating system. However, a balance between stability and mobility must be found because highly stabilizing systems, such as contoured seating or high degrees of tilt in space, can limit the performance of activities of daily living. Stability must be considered in the 3 planes of space and may be provided by all the components of the seating system and the WC. The type of cushion can affect sitting balance [56], and rigid backrests provide higher stability than sling backrests [30]. It is also interesting to note that the word “support” appeared 47 times in the criteria from round one, in relation to neck support, trunk supports, hip support, thigh support, elbow support, knee support and foot support.

Therefore, the stability needs of everyone must be assessed; the challenge for the clinician is to determine the best compromise between mobility and stability. Nevertheless, stability is not often objectively assessed in clinical practice despite the existence of tools that are used in research. Future research should determine reliable methods of assessing the mobility / stability compromise and identify if the type or level of compromise relates to particular postural profiles.

#### Pressure

Pressure was cited by all the panels, with a good level of consensus. It is an issue for a considerable proportion of WC users, including those with DMD [9,11]; some require frequent lifting by caregivers [9]. No studies have evaluated these issues in SMA II. Nevertheless, cutaneous issues, especially redness on the elbows is observed in clinical practice. Pressure injury prevention in the WC involves the use of cushions, power seat functions and active surfaces [17,57]. More than half of the WC experts in this study reported using often or very often the backrest recline, tilt in space and elevated leg rest functions. More than half of those with DMD also used air cushions, which more effectively reduce pressure than gel and foam cushions [58].

The findings of this survey highlight the importance of optimizing the pressure distribution and reducing pressure points in the WC. Interface pressure mapping is widely used to measure pressure distribution and intensity. However, these tools are expensive. If such tools are unavailable, verification of bone protrusions, cushion thickness and the size of the seating device can be helpful.

#### Power seat functions

This keyword was also cited by each panel but was the subject of disagreement between the HP panel and the WC user panels. The mean HP panel importance rating for both SMA II and DMD was high in round two but consensus within this panel was low in rounds three and four for both diseases, despite strong consensus in both WC user panels.

Therefore, a turnaround of opinion occurred in the HP panel between rounds two and three. The Delphi process did not allow replacement of this criterion because it had been selected in round two. This change was unexpected and could be the subject of a future discussion. It is important to note that the majority of the 2 WC user panels had this function on their WC, therefore it had been prescribed by an HP, as it is recommended by international guidelines for DMD and SMA and by practice guidelines for NMD [2,3,25,26]. This finding suggests that HP may hesitate to systematically prescribe the function or may prescribe it at the user’s request but without their own conviction. The price of these more sophisticated WC and the more complex reimbursement process could be a barrier to their prescription [25,26]. Occupational therapists gave lower ratings to power seat functions than the other types of HP. This may be because occupational therapists are closely involved with the funding applications for this type of equipment and are acutely aware of the difficulties and lengthy delays associated with funding. The commercial cost of such WCs ranges from 8000€ to 50000€. In France, the social security system reimburses 3938.01€, which does not cover functions such as tilt in space with large amplitudes or powered leg rests, for example. The reimbursement can be completed by various private, public, or charity funds. The most common is by personal health insurance, which may be paid for by an employer, but not everyone has such insurance cover. The other is by a public fund dedicated to autonomy. However, because of the burdensome administrative process, the individual may wait for 2 to 3 years to receive the funding and be able to buy the chair. As a result, some people choose not to request this funding. Conversely, orthopedic surgeons, who more often identified power seat functions as important criteria, are not prescribers of this type of WC. It is possible that in round 2, power functions received a high rating because of their large clinical benefits, but in round 3, the rating reflected the associated economic issues.

Concerning WC users, 71% of people with SMA II and 86% of people with DMD had a reclining backrest, tilt in space and elevating leg rest functions. More than 50%, particularly those with DMD, used these functions often or very often, especially the reclining backrest and tilt in space (Table 2). The benefits of these power seat functions are recognized by the users who showed an agreement of 93–100% (Table 3). Furthermore, the benefits of powered seating functions have been documented in the scientific literature [17,25,26,59–64]. This information must be disseminated to HP. Although the provision of this type of WC is more difficult, some solutions do exist. For example, associations such as AFM-Téléthon can help to fight these issues through their advocacy actions. AFM-Téléthon can also provide financial support for users with NMD. This information should be better communicated to HP. More discussions between state financial authorities and clinical experts on this topic are necessary to eliminate this significant barrier.

#### Other keywords

“Other” keywords refers to keywords that were not cited by all panels. We do not believe these criteria only apply to one of the diseases, or because of disagreement between HP and WC users, but rather because they were prioritized differently by each panel. Also, some characteristics of SMA II or DMD or associated comorbidities are too heterogeneous for a clear opinion to emerge.

For example, “seat size” was particularly selected in relation to SMA II. Consideration of body size and may be of particular importance in these individuals because of their typically low height and weight [65]. It is not unusual to see people with SMA II lost in a seat that is too large in relation to the size of their pelvis, or with overly long leg rests, or too high a backrest. DMD users may be underweight or overweight, and this should also be taken into account in the choice of seating [66]. Therefore, this keyword could apply for both conditions. This is the same for “headrest”, another keyword, selected by both WC user panels, but not the HP. In both DMD and SMA II, head control is often poor. Therefore the headrest is important to provide support and comfort as well as for safety during the use of power seat functions [9,67]. The headrest supplied with powered WCs may be too basic. More suitable, modular headrests exist, but they are expensive and not well reimbursed. Moreover, the assessment to identify headrest requirements involves complex testing that is not always possible by hospital or technical teams because of lack of knowledge, lack of funding of the assessment time, or lack of test equipment. We believe these results highlight the importance of a suitable headrest to WC users, and that this should not be overlooked by HP.

Therefore, we believe that these keywords, along with “mobility”, “team”, “muscle”, “quality of life”, “upgradability” and “visual”, were not always selected in view of the constraint to select up to 10 criteria; not because they were not important. However, further research is required to verify this.

### Limits

At the time of this study, the definition of an expert could not be framed by certification in France. An inter-university diploma about wheelchair seating was created in September 2021, a few months after the end of the survey. Nevertheless, 3 to 7 years of experience in the field concerned is considered adequate for participation in a Delphi survey [27,68]and the panels of experts had more than 16 years of experience on average.

The attrition rate in WC user panels could have impacted on the results but the personal characteristics of the panels did not change and rates of consensus remained high, therefore, we do not believe this is an issue.

Another limitation of this study was the vocabulary used by the experts, which required expertise to understand. For this reason, we chose not to use word analysis software, and 2 clinical researchers performed the analysis. This was also feasible because the number of criteria suggested in round one was manageable.

From a clinical point of view, these results could be generalized to European and more broadly to all regions of the world if the economic elements are not considered.

## Conclusion

This Delphi study involving HP and wheelchair user experts found similar criteria to optimize WC seating for people with SMA II and DMD. The most important common criteria were comfort, pain prevention, access to the joystick, stability, pressure distribution and power seat functions. Consensus for the 5 first criteria was above 74% across the panels. In contrast, HP and WC user experts did not agree on the importance of power seat functions. The reason for this finding is certainly economic. These results show that wheelchair seating must be considered in terms of abilities or other variables, like environment factors, and not by disease. The identification of 10 WC seating criteria and the similarities between the criteria across HP and WC users with SMA II and DMD provide useful information for both clinicians and researchers. This information can be used to improve healthcare pathways for WC users with NMD. We now plan to confront these results with the opinions of the HP European workshop on wheelchair seating for people with NMD. We also wish to determine a prognostic score to identify the need to modify the WC seating of a given individual, and thus the need to refer the person to a seating advice team.

## Supporting information

S1 File Criteria suggested in round one. (PDF)
