# Supplementary material for: Identification of wheelchair seating criteria in adults with neuromuscular diseases: A Delphi study
Source: PLoS One. 2023 Sep 8;18(9):e0290627. doi: 10.1371/journal.pone.0290627 (PMC10490879; doi:10.1371/journal.pone.0290627)
Supplement: S1 File — (PDF) [file pone.0290627.s001.pdf]

Appendix : Criteria suggested in round one

Question for expert wheelchair users:

Try to identify 6 to 10 factors that, in your opinion, ensure a good static and/or dynamic position in the wheelchair for an adult with your disease.

IMPORTANT: Please formulate the factor according to the following: ‘verb + part of the body OR part of the wheelchair OR principle’. Try to be as precise as possible.

Question for health professionals:

Try to identify 6 to 10 factors that, in your opinion, ensure a good static and/or dynamic position in the wheelchair for an adult with DMD / Try to identify 6 to 10 factors that, in your opinion, ensure a good static and/or dynamic position in the wheelchair for an adult with SMA II.

IMPORTANT: Please formulate the factor according to the following: ‘verb + part of the body OR part of the wheelchair OR principle’. Try to be as precise as possible.

Health professionals for SMA II

1	Stability and overall maintenance of body posture
2	Maintaining balance in the frontal plane
3	Maintaining balance in the sagittal plane
4	Ensure the possibility of movement
5	Compensate for or correct body deformities and contractures (by fitting a moulded seat)
6	Avoid worsening of orthopaedic problems
7	Prevent scoliosis progression
8	Take into account the residual gibbosity
9	Ensure good spinal posture in all planes of space
10	Consider the presence or absence of arthrodesis
11	Ensure a horizontal gaze
12	Have a modular head support
13	Maintain head position and stability
14	Maintain the head and trunk axes
15	Head support with Garchois corset with or without headrest
16	Maintain the head without over constraining; with lateral support if necessary
17	Ensure the head is anterior to the femoral heads
18	Set up that allows visual exploration
19	Head rest fixed to electric wheelchair to compensate for head instability
20	Make a neck support
21	Have a headrest that contacts the occiput
22	Adjust the headrest anterior-posteriorly to avoid pain or discomfort
23	Adapted headrest to maintain cervical spine posture if no corset with neck brace
24	Allow optimal access to the joystick and controls/contactors regardless of the position of the chair
25	Choice of controller according to ability

26	Driving interface that does not cause positioning problems
27	Monitor the position of the joystick in relation to ulnar (wrist) deviation
28	Upgradable wheelchair control adapted to motor ability
29	Adjust the position of the upper limb for maximum driving efficacy
30	Prioritize the choice and position of the joystick (lateral, central, mini, sensitive for permanent adapted access)
31	Facilitate wheelchair driving - adjust the joystick to allow rapid turns
32	Ensure upper limb comfort in all seating positions
33	Maintain and improve upper limb function
34	Support the elbow to ensure a good grip on the joystick (despite wheelchair jolts, changes in position, etc.)
35	Allow a little mobility of the upper limb
36	Place upper limbs on a removable tray table
37	Adjust armrests to balance the trunk and allow better upper limb movement
38	Adjust the armrests to allow elbow flexion for eating
39	Put the armrests or tray table at elbow height
40	Ensure good arm position with adapted and comfortable armrests
41	Stabilise the pelvis
42	Ensure the pelvis is horizontal
43	Prevent pelvic obliquity
44	Compensate for pelvic obliquity in the case of spinal arthrodesis
45	Prevent pelvic anteversion
46	Have an abduction pad between the thighs
47	Stabilise and hold the lower limbs
48	Support the lower limbs well, especially the knees (to prevent swinging of the lower limbs)
49	Support the limbs with appropriate footrests/leg wedges
50	Height adjustable footrests
51	Adjust the footrests (to ensure some weightbearing and to prevent deformities)
52	Place the feet correctly on the rests
53	Encourage weight bearing through the feet as much as possible
54	Prevent lower limb deformities
55	Adapt the position of the lower limbs on the leg rests that come with the wheelchair or made to measure leg rests
56	Stabilise and hold the trunk
57	Mobilise the trunk
58	Stabilise the trunk and support the upper limbs
59	Trunk balanced
60	Line up the trunk
61	Overall trunk balance
62	Hold the trunk, which cannot be supported solely by the chair (+ Garchois corset)
63	The thigh-trunk angle must be adjustable and not fixed at 90°.
64	Trunk supports
65	Maintain trunk mobility
66	Support the thorax using a shoulder or chest harness to compensate for trunk hypotonia
67	Have balanced shoulders

68	Choose postural supports according to the results of a postural assessment (SPCM)
69	Choose suitable postural supports for each body segment
70	Offer electric backrest recline function
71	Offer electric backrest tilt function
72	Offer electric leg rests
73	Choice of motorised functions
74	Offer a multi-position electric wheelchair
75	Offer electric wheelchair seating functions (backrest, seat, leg rest, lift and sit-to-stand)
76	Standing function is essential (to allow weightbearing easily, several times a day)
77	Control posture during standing (wedge, harness, biomechanical backrest, etc.)
78	Enable standing (as long as it provides medical and social benefits)
79	Allow joint mobilisation by activating the electric functions of the wheelchair
80	Memorisation of a reference “comfort” position by the remote control
81	Have a biomechanical backrest
82	Have a comfortable backrest adapted to fixed orthopaedic deformities of the spine
83	Prefer firm seats
84	Mechanically adjust the seat components of the chair (armrest, wedge, feet, backrest, etc.)
85	Stabilise the sitting position
86	Choose a comfortable seat
87	Reposition the buttocks at the back of the chair
88	Take into account the height of the seat, as this type of change is very sensitive
89	Stabilise the entire seat in the wheelchair
90	Check the stability of the proposed seat on varied terrain
91	Cushion with several inflatable zones
92	Choose an air cushion if there is a history of ischial pressure sores or significant ischial pain
93	Use a cushion that is adapted for possible skin problems
94	Use a cushion adapted to the obliquity of the pelvis
95	Distribute the pressure in the seat
96	Vary the distribution of body weight
97	Ensure maximal support surfaces
98	Distribute the support to control the position of the pelvis
99	Distribute pressure under the buttocks
100	Distribute pressure along the entire length of the thighs
101	Relieve protruding parts of the body
102	Distribute pressures of the whole body
103	Distribute support at the back if no brace is used
104	Reduce pressure points
105	Prevent pressure sores
106	Provide comfort and support
107	Provide sufficient comfort
108	Promote comfort through dynamic positioning

109	Wheelchair adapted to the environment (accessibility of the home, vehicle, table, pavement, etc.)
110	Adaptation to clearance heights
111	Control the environment (game consoles, shutters, television, door)
112	Good positioning starts with a good transfer: ensure good first set-up
113	Getting around in an electric wheelchair
114	Ensure mobility during daily activities
115	Take into account the patient's mode of transfer
116	Enable easy transfers with or without technical aids
117	Promote autonomy with the set-up: do not over constrain the person
118	Ensuring the maintenance of autonomy for activities of daily living
119	To encourage the maintenance of autonomy and social participation
120	Facilitate removal from the chair and corset by the physiotherapists
121	To know the person's life habits
122	To know the patient's mode of transfer
123	Evaluate satisfaction, appropriation and quality of life of the wheelchair
124	Know the different wheelchair models
125	Knowing the person's functional and motor deficits
126	Early positioning consultation from the 1st electric wheelchair, then at each change of wheelchair or medical stage (post arthrodesis +++)
127	Carry out a positioning assessment
128	Regular follow-up (physical medicine and rehabilitation doctors, physiotherapists and occupational therapists)
129	Compensate for the various postural disabilities
130	Know whether the patient can move independently or with assistance
131	Thorough assessment of needs using a multidisciplinary approach guarantees a better result
132	Take into account the user's wishes and needs
133	Consider the person's anthropometric measurements when choosing a chair
134	Offer equipment that can be upgraded (adjustments, set-up of additions)
135	Choose a wheelchair adapted to the duration of use
136	Prevent and limit pain (buttocks, lower limbs, etc.)
137	Regularly review and question pain in the sitting position
138	Alleviate pain by lining the seat corset to ensure comfort
139	Carers and relatives must be trained to adjust and handle the equipment
140	Understanding of the importance of good wheelchair positioning by the patient, and adherence to advice
141	Adapt the position of the pelvis using the wheelchair seat
142	Reduce fatigue
143	Facilitate breathing (through dynamic positioning)
144	Improve physiological functions (breathing, digestion, blood circulation)
145	Shock absorption to prevent involuntary changes of position due to uneven terrain
146	Consider the person’s ability to reposition themselves / be repositioned
147	Acceptable overall aesthetics for the patient
148	Offer supports for ventilation, oxygen cylinder and electric aspirator
149	Ensure an easy transition from motorisation mode to manual mode
150	Ensure the autonomy and charging of the wheelchair
151	Upgradability

Health professionals for DMD

	Criteria
1	Stability and overall maintenance of body posture
2	Maintain balance in the frontal plane
3	Maintain balance in the sagittal plane
4	Avoid worsening of orthopaedic problems
5	Prevent progression of scoliosis
6	Do not overcorrect kyphosis in the sitting position
7	Take into account the residual gibbosity
8	Ensure good spinal posture in all planes of space
9	Offer concave-shaped thoracic support and opposite-shaped lumbar support
10	Compensate for the lumbar lordosis with a lumbar support of appropriate thickness for the lordosis
11	Take into account the presence or not of an arthrodesis
12	Ensure horizontal gaze
13	Allow visual exploration
14	Support the head without constraining it using lateral supports if necessary
15	Support and stabilise the head
16	Position the external auditory canal above or behind the femoral heads
17	Include a modular headrest
18	Include a neck brace
19	Headrest in contact with the occipital region
20	Cervical-head position that allows swallowing
21	Prevent cervical spine deformations
22	Align the head and trunk
23	Adjust the headrest anterior-posteriorly to avoid pain or discomfort
24	Allow optimal access to the joystick and controls/contactors from any position of the chair
25	Driving interface that does not cause positioning problems
26	Upgradable wheelchair control adapted to motor ability
27	Choose and position the joystick after adapting the wheelchair seat
28	Adapt the wheelchair control so that the driver does not have to adopt a bad position
29	Position the upper limb for maximum driving efficiency
30	Choose the control according to ability
31	Facilitate driving of the wheelchair
32	Maintain driving ability in the power wheelchair
33	Support the elbow to ensure a good grip on the joystick (despite wheelchair jolts, changes in position, etc.)
34	Support the upper limbs
35	Ensure upper limb comfort in all seating positions
36	Free the upper limbs
37	Maintain upper limb function
38	Allow some upper limb mobility
39	Place upper limbs on a removable tray table
40	Offer an upper limb compensation device in addition to the wheelchair
41	Adjust the armrests to balance the trunk and improve upper limb movement
42	Have the armrests at the right height for the elbows

43	Ensure good arm position by using adapted and comfortable armrests
44	Stabilise the pelvis
45	Prevent pelvic obliquity
46	Distribute pressures at the pelvis. Make the pelvis horizontal
47	Lumbopelvic region in contact with the back of the chair
48	Stabilise the lower limbs
49	Choose the degree of abduction
50	Improve and stabilise lower limb supports (to prevent deformities)
51	Height adjustable foot rests
52	Prevent the development of ankle deformities
53	Position the lower limbs to prevent rubbing/pressure from the footrests
54	Adapt the position of the lower limbs to their posture in the 3 planes of space
55	Encourage plantar weight bearing as much as possible
56	Orthopaedic shoes if foot and ankle support is difficult
57	Stabilise the trunk
58	Improve the comfort of the trunk
59	Improve the contact surface of the trunk
60	Ensure frontal balance with trunk wedges
61	Encourage trunk mobility to vary pressure points
62	The thigh-trunk angle should be adjustable and not fixed at 90
63	Prevent trunk hyper flexion
64	Have balanced shoulders
65	Support the thorax to compensate for trunk hypotonia using a shoulder or chest harness
66	Choose postural supports according to the results of a postural assessment (SPCM)
67	Select a suitable postural support for each body segment
68	Choose reliable posture supports that are easy to set-up and remove to facilitate transfers
69	Offer electric tilting seat
70	Offer electric tilting backrest
71	Offer electric leg rests
72	Offer standing function (with knee, hip and chest control)
73	Control posture during standing (wedge, harness, biomechanical backrest, etc.)
74	Offer a multi-position power wheelchair
75	Offer electric wheelchair seating functions (backrest, seat, leg rest, lift and sit-to-stand)
76	Choice of motorised functions
77	Allow joint mobility by activating the electric functions of the wheelchair
78	Memorise a reference “comfort” position using the remote control
79	Know the limits of use of the electric seat functions (depending on the patient's discomfort, fear and loss of motor ability)
80	Maintain posture when changing position with the electric backrest and seat functions
81	Comfortable backrest adapted to fixed orthopaedic deformities of the spine (kyphosis, scoliosis)
82	Have a biomechanical backrest
83	Adjust the seat according to anthropometric measurements

84	Mechanical adjustment of chair seat components (armrest, footrest, backrest, etc.)
85	Prefer firm seats
86	Stabilise the sitting position
87	To choose a comfortable seat
88	Check the stability of the proposed seat (when moving on uneven ground)
89	Choose the right seat and wheelchair to allow good access without obstacles
90	Choose the right density of foam
91	Choose a cushion adapted to anthropometric measurements
92	Chose a cushion that will prevent skin issues
93	Choose an appropriate cushion for skin issues
94	Adapt the shape of the cushion to the postural needs of the pelvis
95	Multi-zone air cushion
96	Choose an air cushion if there is a history of ischial pressure sores or significant ischial pain
97	Use a cushion adapted to the obliquity of the pelvis
98	Distribute the pressure over the seat
99	Vary the pressure distribution
100	Ensure maximum support surfaces
101	Distribute pressures along the entire length of the thighs
102	Relieve the protruding parts of the body
103	Distribute all body contact points
104	Distribute pressures on the back
105	Reduce pressure points
106	Quality of support: firm and on large surfaces
107	Prevent pressure sores
108	Ensure comfort to allow long sitting durations
109	Promote the comfort of the person
110	Promote the person's comfort through dynamic positioning
111	Find a compromise between comfort and function
112	Choosing a wheelchair that will provide comfort
113	Wheelchair adapted to the environment (accessibility of the home, vehicle, table, pavement, etc.)
114	Environment control (video game consoles, shutters, door, television)
115	Guarantee the maintenance of autonomy in the activities of daily living and social life
116	Promote autonomy with the set-up: Do not overly constrain the person
117	Compromise between prevention of deformities and trunk mobility
118	Allow some mobility for life activities
119	Getting around in an electric wheelchair
120	Early positioning consultation from the 1st electric wheelchair, then when changing wheelchair or medical stage (post arthrodesis +++)
121	Choose a wheelchair adapted to life habits
122	Choose a wheelchair adapted to daily duration of use
123	Choose a wheelchair adapted for transfers
124	Ensure compatibility with the patient's usual transfer mode
125	Allow easy transfers with or without technical aids
126	Good positioning begins with a good transfer: ensure a good initial set-up
127	Know the person's lifestyle

128	Know the patient's transfer mode
129	Know the different chair models
130	Know the person’s functional and motor impairments
131	Regular follow-up (physical medicine and rehabilitation doctor, physiotherapist and occupational therapist)
132	Perform a positioning assessment
133	Compensate for different postural disabilities
134	Determine whether the patient can move independently or with assistance
135	Take into account the user's wishes and needs
136	Take into account the patient's feelings of insecurity when certain positions are changed
137	Evaluate satisfaction, appropriation and quality of life in the wheelchair
138	Know if the patient has bone fragility
139	Thorough assessment of needs using a multidisciplinary approach to guarantee a better result
140	Possibility to adjust and adapt the wheelchair
141	Choose a wheelchair adapted to anthropometric measurements
142	Limit pain (buttocks, back, lower limbs)
143	Prevent pain
144	Understanding of the importance of good wheelchair positioning by the patient, and adherence to advice
145	Train family and friends to adjust and handle the equipment
146	Reduce fatigue
147	Agree acceptable changes with the patient considering knowledge of the progression of the pathology and its consequences
148	Shock absorption to prevent unintentional changes of position on uneven terrain
149	Acceptable overall aesthetics
150	Improve physiological functions (breathing, digestion, blood circulation)
151	Facilitate breathing (through dynamic positioning)
152	Ensure autonomy and charging of the wheelchair
153	Offer support for ventilation, oxygen cylinder and electric aspirator
154	Adapt the position of the pelvis using the wheelchair seat
155	Ensure an easy transition from motorisation to movement mode
156	Allow for upgradability
157	Maintain abilities
158	Facilitate movement

Wheelchair users with SMA II

1	Ensure proper positioning of the thighs and hips to avoid inflammation of the sciatic nerve
2	Reduce pressure under the buttocks, thighs, neck and lumbar areas
3	Have a seat cushion adapted to the morphology
4	Good stability of the seat cushion is essential
5	Have an adequate and comfortable cushion to be able to stay in the chair all day without buttock pain
6	Have a cushion under the buttocks that does not cause pain even over a long period of time and that guarantees stability
7	Have a comfortable cushion for straight positioning of the trunk
8	Have a positioning cushion with small cushions that can be moved to relieve certain pressure points and prevent pressure sores
9	Memory foam cushions are a good in-between, leather and air cushions
10	It is essential that the backrest and seat are comfortable to avoid pain when sitting in the chair
11	Do not have pressure more on one side of the seat than the other
12	Position the lower back at the back of the seat with a 1cm margin between the back and the back of the chair to avoid friction and facilitate movement
13	Use adjustable and removable thigh supports (no tools required)
14	Be positioned straight and well supported on the sides using "trunk wedges" so as not to tip over to one side because of lack of tone and scoliosis and also to avoid moving when using the standing function
15	To have a rear seat tilt that allows a really comfortable resting position, without pain or to help to be well positioned at the back of the chair seat or to cross pavements more easily
16	To be able to tilt the seat without blocking the driving (safety)
17	Use the seat tilt regularly enough during the day to relieve the lumbar region
18	Keeping the trunk as straight as possible (to make it easier to perform certain daily activities involving the upper limbs and avoid collapse of the rib cage)
19	Have a stable chest
20	Have a backrest that fits your back, especially if you have a big lordosis
21	To have a backrest that provides support and security without restraining neck movements
22	To have an ergonomic seat back that provides optimum support regardless of the sitting position
23	Have a backrest of the same brand as the cushion (the vicair with air-cells) to be able to support all parts of the back
24	Have a more adjustable backrest height that is easy to adapt to the size of the back
25	Leave about 5 to 10 cm between the shoulders and the back of the chair to encourage an active position (working, eating)
26	Stick the shoulders to the backrest and put the head on the headrest for resting, in the car
27	Have a backrest cushion adapted to your morphology
28	Have a trunk support, possibly with trunk wedges
29	Support the trunk well so that the position does not slip

30	Before using trunk wedges, first try a slightly curved backrest, which keeps the back straight without feeling blocked
31	Have trunk wedges, pelvic wedges and thigh wedges that promote stability in any seating configuration
32	Have an electric reclining backrest (to facilitate mobilisation in the chair, to relieve pain, to relax, to change position, to adapt to each environment, to relieve the position, facilitate certain movements, to rest the back)
33	Slightly tilt the back of the chair and lean against it (back + head)
34	Use the electric reclining function of the backrest to be able to move the hips a little during the day
35	Have a biomechanical backrest that allows the head to be supported regardless of the chair back tilt and avoids the need to constantly readjust the wedges/cushions that move when this function is not available
36	Be well equipped with a harness/belts for safety
37	Avoid harnesses which are often too heavy, compress the rib cage and prevent upper limb movement. Prefer small chest strap with velcro
38	Being able to lie down as flat as possible would still be a bonus with the tilt function of the backrest
39	Be able to lie down or almost lie down in the chair in case of respiratory problems, to pee, to change clothes or even to sleep using the tilt functions of the backrest and the leg rests
40	Have a lift to be at the same height as able-bodied people
41	Have the legs well aligned with the hips
42	Ensure that both knees are at the same level
43	Legs should be relatively centred, in line with the body (about 15 cm between the knees)
44	Have an electric leg-lift (to facilitate mobilisation in the chair, to relieve pain, to allow relaxation)
45	Relieve the knees with electric leg rests
46	Make sure that the feet are well supported on the pallet, otherwise raise it until they are. This will help with balance for positioning in the chair
47	Have small footrests for small feet
48	Support the feet with the footrests without too much pressure
49	Have the feet flat with the footrests set at the right height
50	Have your feet placed on something comfortable so that you can remain barefoot
51	Have the foot plates a little forward so that they don't pull behind the ankles
52	Place your feet flat on the footplate and in line with your knees (feet 13 / 15 cm apart)
53	Footrests help to stabilise the feet and legs when manoeuvring the chair
54	Have retractable footrests. Folding them down is not comfortable for the legs when they are folded inwards
55	Have separate footrests for better and more precise adjustment
56	Momentarily remove the feet from the footrest by putting them on either side to relieve ankle and leg muscles
57	Be able to change positions to avoid pressure points, to relieve pain and facilitate breathing, independently using a seat tilt function, electrically reclining backrest and electric leg rest

58	Use a multi-position chair +++ (reclining backrest; lift; seat tilt; electric leg rest)
59	Be able to change position easily and without help thanks to the joystick
60	Stand up once a day to relax the muscles, as a form of rehabilitation
61	Adjust the lower leg supports and program the chair for a really comfortable standing position
62	Program standing in the joystick to stand without any problems
63	Have knee pads in place for standing and to keep the legs abducted
64	Find the right balance between body support (especially for the trunk) and freedom of movement in order to continue to use muscles that are still functional
65	Have good head support (to avoid cervical trauma and to be more comfortable in certain daily activities and driving)
66	Have a suitable headrest
67	Have a good headrest (chair or brace) so that the head is well positioned throughout the day, to ensure swallowing, avoid pain and pressure sores, and avoid choking
68	Have a headrest that is easy to adjust
69	Make sure that the horizon is rather straight. If not, choose the headrest according to the person's head control and their level of fatigue
70	Have headrest adjustments (to facilitate comfort and stability, safety for driving in the wheelchair in case of chaos on the road (the head should not move in all directions), safety in the car
71	Have a headrest to rest your head on in case of fatigue
72	Be well supported to be safe during transport or in chaotic places (belt, trunk support, neck and head support or even a headband)
73	Be stable so that you can drive without fear of holes in the pavement, stones, grass, etc.
74	Achieve the necessary stability in the wheelchair for optimal propulsion and function, but also to distribute the weight and avoid pressure sores
75	To prevent the hips from slipping, have a support that blocks the hips during the day when crossing a pavement, paving stones, gravel, etc.).
76	Have very good shock absorbers for going outside without being shaken in case of head instability
77	Do not feel the shocks too much thanks to good shock absorbers and air wheels
78	Avoid as much as possible leather armrests, seats and backrests (leather does not help to avoid pressure, especially as it becomes very hot in the sun)
79	Use gel armrests!
80	Adjust the armrests and the joystick to the right height/depth to facilitate comfort and stability of the trunk, to avoid spinal deformities and to be able to drive the chair
81	Have a good arm position that allows driving (the elbow must be supported without pain and the armrest must not block or hinder the mobility of the arm to allow safe and autonomous driving)
82	Have comfortable armrests to avoid elbow pain
83	Have the elbows resting on the armrests to relax the shoulders
84	Good positioning is the key to avoiding sudden falls during outdoor trips

85	Have a tray table to have what is needed at hand
86	Have a chair of the right size for your height

Wheelchair users with DMD

1	Having the right size seat for comfort
2	Choosing the right cushion for a good seating comfort
3	Having a cushion to avoid pressure sores
4	Being in the middle of the cushion
5	Having the right type of cushion to avoid buttock pain
6	Have a foam cushion for a better pelvic stability
7	Have a straight and balanced pelvis (start the positioning by checking the pelvis first before dealing with any other part of the body)
8	Have symmetrical support on both buttocks
9	Be positioned at the back of the seat
10	Have the buttocks at the back of the cushion when sitting in the chair but not so far back that you lean forward
11	Maintain the position at the back of the chair with a lap belt in addition to the harness, which is used for movement
12	Be well supported with pelvic, thigh and trunk supports, etc. if necessary
13	Position the hips with thigh wedges and hip wedges (thigh wedges prevent uncomfortable spreading of the legs and hip wedges keep the hips well in the centre of the buttock cushion)
14	Have thigh wedges to keep the legs in line with the pelvis
15	Tighten the belt, but not too much, so that the back does not fall forward, and the back is well supported by the backrest
16	Have a seat tilt function to change positions to avoid pressure points that can lead to skin lesions and pressure sores
17	Use the seat tilt often
18	Use the seat tilt to relieve body weight
19	Have a forward seat tilt for eating (to avoid choking and to be able to swallow food properly)
20	Have an electric seat tilt
21	Be able to lean forward easily to relieve pressure
22	Position the pelvis, hips and buttocks comfortably in relation to the cushion, which should allow a good position without too much pressure. In addition, have a seat that varies the pressure and allows regular movement
23	Have a made to measure backrest for good lateral trunk support to avoid leaning/slouching, which can make you lose the joystick
24	Position the back in relation to the backrest, which must be comfortable enough to be leaned on without pain over time, to be well supported, limit muscle fatigue and avoid back problems
25	Have a backrest that is the right width and height for comfort and support
26	Regularly rest your back against the backrest and your neck against the headrest to avoid staying in a bad position for too long and thus avoid pain
27	Have a backrest that makes an angle of 94° with the seat (when the backrest makes an angle of 90° with the seat, the position is very uncomfortable)
28	Use trunk wedges or a wrap-around backrest to ensure great stability
29	Support the chest laterally using trunk wedges to ensure an optimal and comfortable position.

30	Have a chair with 1 or even 2 trunk wedges to be really straight
31	Do not lean to one side
32	Pull the buttocks well to one side and the other to make sure the back is as straight as possible and avoid leaning when being positioned in the chair
33	Having a harness for going outside is more comfortable and safer
34	Choosing the right model of seat belt or harness
35	Have an electrically reclining backrest to be able to change position regularly
36	Have an electrically reclining backrest
37	Have your head against the headrest
38	Position the head and neck at the level of the headrest to make sure it is well supported on all sides. The headrest should be sufficiently far forward to allow good breathing and swallowing. But not too far forward so as not to strain the neck.
39	Adjust the depth of the headrest so that the back is well supported by the backrest.
40	The position of the control (screen) and the joystick must allow a good position of the head, hand and fingers
41	Have a well-adjusted headrest to be properly supported
42	Have a headrest of the correct size and foam to help hold the head and for comfort
43	Have the legs aligned for better stability, weight distribution and to limit the risk of pressure sores
44	Knees at a 90 degree angle
45	Knee braces to keep the legs parallel
46	Have knee supports on the chair for good support and to avoid straps (especially when driving in case of jolting, you may find yourself badly positioned)
47	Knee supports so that you can see if you are sitting in the back of the chair
48	Legs and feet should be in line and well supported
49	Have leg rests to prevent the feet from sliding backwards
50	Adjust the height of the footplates so that the feet are supported but not too much (the thighs should rest on the cushion so that the knees are not too high)
51	Have your feet flat on the footrests
52	Adjust the height of the footplates so that the feet are touching but not too much
53	Raise the footrests to avoid numbness in the legs
54	Extend the length of the footrests and spread them out to position the feet properly and avoid having the legs too tight
55	Extend the length of the foot plates and spread them out to position the feet properly and avoid having the legs too close together
56	Use electric footrests
57	Use electric leg lifts to stretch the legs several times a day
58	Adjust the armrests so that the shoulders are relaxed and the muscles are not overstretched
59	Have your arms resting on the armrests to prevent shoulder muscle pain at the end of the day and to improve stability
60	Have armrests to facilitate driving and support

61	Do not have armrests that are too hard to avoid injuring elbows and/or arms and to avoid pressure sores
62	Be able to adjust the armrests independently of each other
63	Have a removable tray table on the chair for meals and to be able to read and look at your mobile phone with your head upright
64	Have a tray table so that you can put your hands on it
65	Have the hands resting flat to limit contractures in the hands and wrists
66	Provide foam or a specific cushion to put the hands on the tray table
67	Have the option of a lift to raise the chair to the right height
68	Have a stand-up chair
69	Have a headrest that moves forward when standing (if the headrest stays in the same place when standing, the head moves backwards which is very uncomfortable)
70	Use the stand function or backwards seat tilt at least once a day
71	Have a seat and backrest that tilt
72	Regularly change your position in the chair by using the seat tilt and back tilt functions
73	Regularly change position (lie down, extend legs, etc.)
74	Find the right compromise between static and dynamic positioning (having to change position regularly can be restrictive. A position that is too static can also cause problems in the long term)
75	Respect each step of the set up and the positioning of each element in order to limit future pain
76	Strike a balance between the "ideal" position and the acceptability of the proposed improvements (improvements in positioning should not prevent the performance of daily activities)
77	To be surrounded by competent health professionals to get the best advice and improve the WC set-up
78	Regularly check the various adjustments, seat positioning, wear and tear of the material, plan for foam changes if necessary
79	Have your hand very precisely positioned on the joystick
80	Have easy access to the controls and switches through good positioning of the arms and hands
81	Be able to position your hand easily on the joystick
82	Easy access to the joystick without having to exert too much effort
